# Deconstructing the Dimensions of Mycobiome Fingerprints in Luohandu Cave, Guilin, Southern China

**DOI:** 10.3390/microorganisms12010211

**Published:** 2024-01-20

**Authors:** Bai-Ying Man, Xing Xiang, Xiao-Yu Cheng, Hong-Mei Wang, Chun-Tian Su, Qi-Bo Huang, Yang Luo, Chao Zhang, Gang Cheng, Yu-Yang Ni, Xing-Hua Shao

**Affiliations:** 1 College of Life Science, Shangrao Normal University, Shangrao 334001, China; xiangxing1989@126.com (X.X.); yangl1829848@163.com (Y.L.); zhangchao_20180604@163.com (C.Z.); enjoy123fly@163.com (G.C.); 312144@sru.edu.cn (Y.-Y.N.); xinghuashao@126.com (X.-H.S.); 2 Key Laboratory for Regional Plants Conservation and Ecological Restoration of Northeast Jiangxi, Shangrao Normal University, Shangrao 334001, China; 3 School of Environmental Studies, China University of Geosciences, Wuhan 430074, China; chengxy@cug.edu.cn; 4 State Key Laboratory of Geobiology and Environmental Geology, China University of Geosciences, Wuhan 430074, China; 5 Institute of Karst Geology, CAGS/Key Laboratory of Karst Dynamics, MNR & GZAR, Guilin 541004, Guangxi, China; schuntian@mail.cgs.gov.cn (C.-T.S.); qbohuang0108@163.com (Q.-B.H.); 6 Pingguo Guangxi, Karst Ecosystem, National Observation and Research Station, Pingguo 531406, Guangxi, China

**Keywords:** subterranean karst ecosystem, Luohandu cave, Illumina Hiseq sequencing, mycobiomes, co-occurrence network

## Abstract

Subterranean karst caves are windows into the terrestrial subsurface to deconstruct the dimensions of mycobiome fingerprints. However, impeded by the constraints of remote locations, the inaccessibility of specimens and technical limitations, the mycobiome of subterranean karst caves has remained largely unknown. Weathered rock and sediment samples were collected from Luohandu cave (Guilin, Southern China) and subjected to Illumina Hiseq sequencing of ITS1 genes. A total of 267 known genera and 90 known orders in 15 phyla were revealed in the mycobiomes. Ascomycota dominated all samples, followed by Basidiomycota and Mortierellomycota. The sediments possessed the relatively highest alpha diversity and were significantly different from weathered rocks according to the diversity indices and richness metrics. Fifteen families and eight genera with significant differences were detected in the sediment samples. The Ca/Mg ratio appeared to significantly affect the structure of the mycobiome communities. Ascomycota appeared to exert a controlling influence on the mycobiome co-occurrence network of the sediments, while Ascomycota and Basidiomycota were found to be the main phyla in the mycobiome co-occurrence network of weathered rocks. Our results provide a more comprehensive dimension to the mycobiome fingerprints of Luohandu cave and a new window into the mycobiome communities and the ecology of subterranean karst cave ecosystems.

## 1. Introduction

Fungi, a highly diverse group of eukaryotic organisms distinct from plants, animals and protist, exert crucial roles across all ecosystems on Earth. As the second largest kingdom of eukaryotic life, fungi are widely distributed and exhibit a pattern of regional endemism on a global scale [1]. Although community diversity and new functional groups of mycobiomes in aquatic and terrestrial ecosystems are constantly being revealed, which is facilitated by technological advances from culture to non-culture approaches, only a fraction of these mycobiome constituents has been discovered in comparison to the estimated 1.5–5.1 million species [2,3]. This is particularly true for subterranean environments such as karst caves, which serve as reservoirs for specialized fungi, despite the hostile environment that prevails in the absence of light and in the presence of high mineral concentrations, high humidity and a low organic carbon input [4]. As typical extreme subterranean environments with spatial heterogeneity of the cave microbiome [5], such sites may hold an incredible “dark fungal diversity”, which is still underestimated.

Despite the study of cave fungi having a history of 230 years to date, 91.5% of the research works in this field still rely on culture-dependent methods [6,7,8,9]. With breakthroughs in the bottleneck of DNA extraction from cave rock samples and high-throughput meta-barcoding revolutionizing our understanding of global fungal diversity and function, much of the previously “hidden diversity”, new taxa and previously unknown fungal lineages are continuously revealed in subterranean karst caves [7,10,11,12,13,14,15,16]. Although the results of these studies constantly increase our understanding of the genetic diversity, ecology and evolution of fungi, improving mural preservation and drug discovery, cave-dwelling fungi still have received far less attention than prokaryotes [4,5,17]. Previous studies showed that karst caves are populated predominantly by rock-inhabiting fungi of two classes of Ascomycota: Dothideomycetes and Eurotiomycetes [18,19]. Furthermore, the high abundance of Sordariomycetes, another class of Ascomycota, was revealed in cave-weathered dolomite rocks, mainly of the order of Hypocreales, via culture-dependent methods coupled with high-throughput sequencing [4,7]. The aforementioned three dominant classes of Ascomycota were also reported in a study by Satinee et al. in two karst caves in Thailand [8]. Ascomycota are a dominant phylum, and the common genera *Aspergillus*, *Fusarium*, *Penicillium* and *Trichoderma* are frequently founded in karst caves [6,7,16]. On the other hand, caves provide an ideal system for examining the pattern of regional endemism of fungi due to the geographical isolation and typical climate in a given region, which strongly affect the diversity and ecology of cave-dwelling fungi. As a typical subterranean isolation ecosystem, caves have diverse environments (rocks, sediments, bat guanos, dripping water, air, etc.) differing in both physical and chemical composition, whilst the composition and community structure of mycobiomes are further influenced by microclimatic conditions, substrate and spatial variables, in addition to human activities, and significantly differ in different niches [4,20,21,22].

The subterranean karst cave ecosystem is relatively underexplored in Guilin city, Guangxi province, south-western China. This is true for its microorganisms, especially in relation to mycobiome diversity in cave environments. Luohandu cave, a pristine karst cave in Guilin City, China, was only studied by Yang et al. for the environmental driving mechanisms and community assembly process of bacterial communities [23] and upland soil cluster gamma (USCγ) groups [24]. In contrast, its mycobiome has yet to be characterized. Therefore, the aim of this work was to (1) investigate the mycobiomes of weathered rocks and sediments via the high-throughput sequencing technology; (2) reveal the correlations between the structure of the mycobiomes and environmental variables; (3) deconstruct the dimensions of the mycobiome fingerprints of Luohandu cave.

## 2. Materials and Methods

### 2.1. Cave Description and Sample Collection

**Figure 1:**
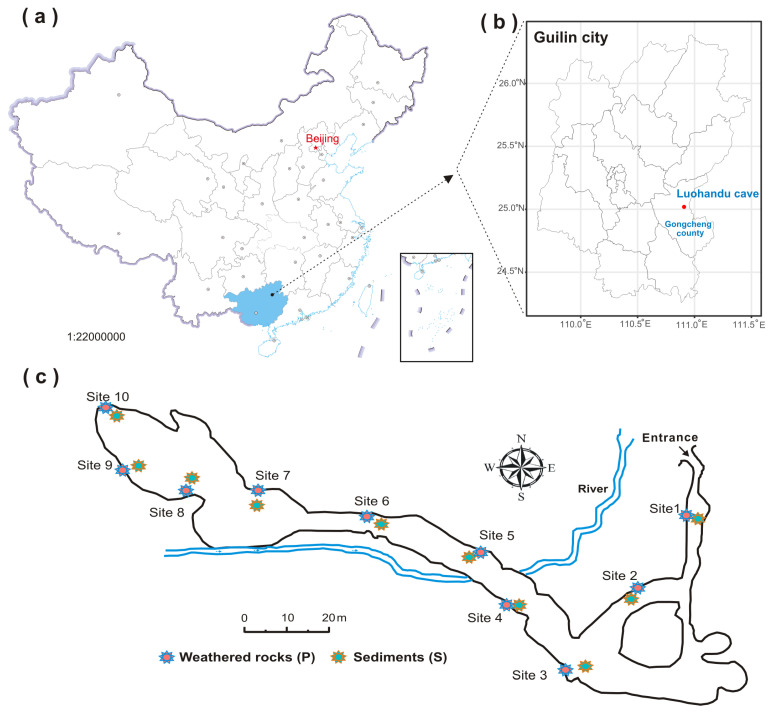
Location of Luohandu cave and the sampling sites (modified from [24]). (**a**) The colored aera shows the location of Guangxi province, China. (**b**) The location of Luohandu cave in Guilin city. (**c**) Sampling sites of weathered rocks (P) and sediments (S).

Luohandu cave (25°0′55.8″ N~110°54′14.2″ E) is remotely situated on the east of Baiyan village, Jiahui township, Guilin city, Guangxi province, southern China (Figure 1a,b). It developed in the Donggangling Formation of in Middle Devonian and is overlain by ~136 m of dolomite [24]. The cave is about 356 m long, 2~12 m in height and 4~25 m in width, with a sole entrance 232 m above the foot of karst peaks. The subtropical monsoon climate exerts a significant influence on this karst region. The mean temperature is 19.21 ± 0.51 °C, and the mean annual precipitation is 1453 mm. The average annual relative humidity is 74%. The cave interior is dark all the year round and less affected by human activities.

Ten sampling sites were selected from the entrance to the end of the Luohandu cave (Figure 1c). Triplicate samples of weathered rocks (P, P1~P10, n = 30) and sediments (S, S1~S10, n = 30) were collected aseptically in each sampling site in 50 mL sterile plastic centrifuge tubes (Corning) by the five-point diagonal sampling method. The samples were transported on ice to the environmental microbiology lab at Shangrao Normal University and stored at −80 °C for further use. Freeze-dried and sieved samples were mixed with ultrapure water (1:5, wt/vol), vortexed for 10 min, centrifuged at 6800× *g* for 10 min and then filtered using a 0.22 μm membrane for physicochemical analysis, including the measurement of pH, TOC (total organic carbon) and the concentration of anions and cations. TOC content, anions and cations were analyzed by a C-S analyzer (EA 4000, Analytik Jena AG, Jena, Germany), anionic chromatography (ICS-600, Thermo Scientific, Waltham, MA, USA) and ICP-OES (iCAP 7, 600+, Thermo Scientific), respectively. The pH was measured by a multi-parameter water quality detector (HACH, Loveland, CO, USA) [25].

### 2.2. DNA Extraction and Mycobiome Sequencing

We used 1 g of freeze-dried samples of weathered rocks and sediments for total nucleic acid extraction with the PowerSoil DNA Kit (MoBio Laboratories, Inc., Carlsbad, CA, USA). The ITS1 region of the fungal rRNA gene was amplified using the primers pairs ITS5–1737F and ITS2–2043R [4]. For the PCR reactions, a total volume of 50 μL contained 25 μL of Premix Taq (Takara Biotechnology, Dalian Co., Ltd., Dalian, China), 1 μL each of the tag-encoded primers (10 mM), 3 μL of DNA template (20 ng/μL) and 20 μL of RNAase-free water. PCR amplifications were performed in triplicate under conditions described previously [7]. The PCR products were qualified by a Nanodrop 2000 spectrophotometer (ND 2000; Thermo Scientific, USA) and visualized by 2% agarose gel electrophoresis. Negative controls were processed in parallel to avoid possible contamination during the experiment. Libraries of the samples were generated using TruSeqTM DNA Sample Prep Kit (Illumina, San Diego, CA, USA) following the manufacturer’s instructions and assessed on the QuantiFluor™-ST system (Promega, Madison, WI, USA). Mycobiome sequencing was performed on the Illumina Hiseq 2500 platform at Shanghai Majorbio Bio-Pharm Technology Co. Ltd. (Shanghai, China). The raw data were deposited in the National Omics Data Encyclopedia (NODE; https://www.biosino.org/node, accessed on 21 December 2023) under the project numbers OEP004875 and OEP004876.

### 2.3. Bioinformatics Analysis

The raw paired-end data were subjected to de-multiplexing based on the barcode sequences of each sample and then analyzed by the dada2 plugin of QIIME2 software (version: 2020.11) [26]. The plugin integrated the analysis by performing filtering, de-replication and chimera removal and by clustering sequences at the amplicon sequence variant (ASV) level. Taxonomic information of the obtained ASVs was gathered using the classification sklearn algorithm of the qiime2-feature-classifier [27] plugin from the UNITE fungal ITS database version 9.0 [28]. The downstream analysis was conducted in R environment (4.3.1). Diversity indices, such as community richness (observed species, Chao1, ACE), community diversity (Shannon, Simpson) and phylogenetic alpha metrics (Faith’s PD index (PD), net nearest taxon index (NTI), net relatedness index (NRI)) were calculated by the phyloseq (version: 1.46.0) and MicrobiotaProcess (version: 1.14.0) packages. Differences in alpha diversity for all pairwise differences between means were compared by nonparametric statistical tests (Wilcox test). Beta diversity analysis was performed to explore the differences in mycobiome composition at the various sampling sites. The analysis was performed based on the Aitchison distance metric, which was calculated by applying a centered log-ratio transformation to the phylotype count table using the ALDEx2 package (version: 1.34.0). The resulting dissimilarity matrix was then visualized using principal component analysis (PCA) via the ggplot2 package (version: 3.4.4). Differential taxa at the family and genus levels between the two groups were identified using Welch’s *t*-test and Wilcoxon test with the microeco package (version: 1.3.1). To account for multiple comparisons, the Benjamini–Hochberg false discovery rate (FDR) correction was applied. Statistical significance was determined at a significance level of *p* < 0.05, indicating a significant difference between the compared groups. To present the differences in the mycobiomes at the taxonomic level, a heat map was created with MicrobiotaProcess. The Mantel test was employed to assess the correlation between the mycobiome community and environmental factors. The test was performed and visualized using the linkET package (version 0.0.7.4).

The ASVs in sediments and weathered rocks were used to construct co-occurrence networks. To optimize the networks’ specificity and sensitivity, the ASVs were filtered based on abundance (>0.05%) and frequency (>20% of samples); the remaining ASVs (219 vs. 107) were used to calculate the Spearman rank correlations (rho). Significant relations with rho > 0.663 (sediments) and >0.641 (weathered rocks), with *p* < 0.01, which were determined by the random matrix theory-based approach using the RMThreshold (version:1.1) package [29], were selected to construct co-occurrence and mutual-exclusion networks using the meconetcomp (version: 0.4.1) package [30] and visualized by Gephi software (version: 0.9.2). In the co-occurrence networks, the nodes represent the mycobiome ASVs, and the edges reflect significant correlations between them. The meconetcomp package was used to determine the topological properties of the co-occurrence networks, including betweenness centrality, closeness centrality, degree at the node level, modularity, average path length and network diameter at the network level. Nodes that exhibit the highest degree and the highest closeness centrality and the lowest betweenness centrality are commonly recognized as keystone species. In the weathered rock network, ASVs with degree >5, closeness centrality <0.39 and betweenness centrality <16 were identified as keystone species. In contrast, for the sediment network, keystone species were selected based on the thresholds of 16 for node degree, 0.36 for closeness centrality and 12 for betweenness centrality. Moreover, subnetworks were extracted for individual samples within the weathered rock and sediment networks based on node occurrence. Subsequently, Spearman correlation between the topological indexes of the subnetworks and environmental factors was calculated and visualized using the meconetcomp (version: 0.4.1) package in the R environment.

## 3. Results

### 3.1. Physicochemical Properties of Weathered Rocks and Sediments in Luohandu Cave

The mean temperature of air was 19.21 ± 0.51 °C. The TOC of weathered rocks was relatively higher than that of the sediments, with mean values of 3.86 ± 2.24 and 1.22 ± 1.05, respectively. The samples of weathered rocks and sediments were slightly alkaline, with pH mean values of 8.72 ± 0.23 and 8.77 ± 0.68, respectively. The sediments had lower concentrations of K^+^ (mean value, 0.45 ± 0.35 mg/L) compared with weathered rocks (1.26 ± 2.34 mg/L). The SO_4_^2−^ concentration in the sediments, with a mean value of 3.22 ± 4.03 mg/L, was also lower than in weathered rocks (7.35 ± 13.30 mg/L). The Ca/Mg ratio was higher in weathered rocks, with a mean value of 68.62 ± 27.02, than in the sediments (4.22 ± 17.41).

### 3.2. Mycobiome Community Structure and Interaction with the Environmental Conditions

**Figure 2:**
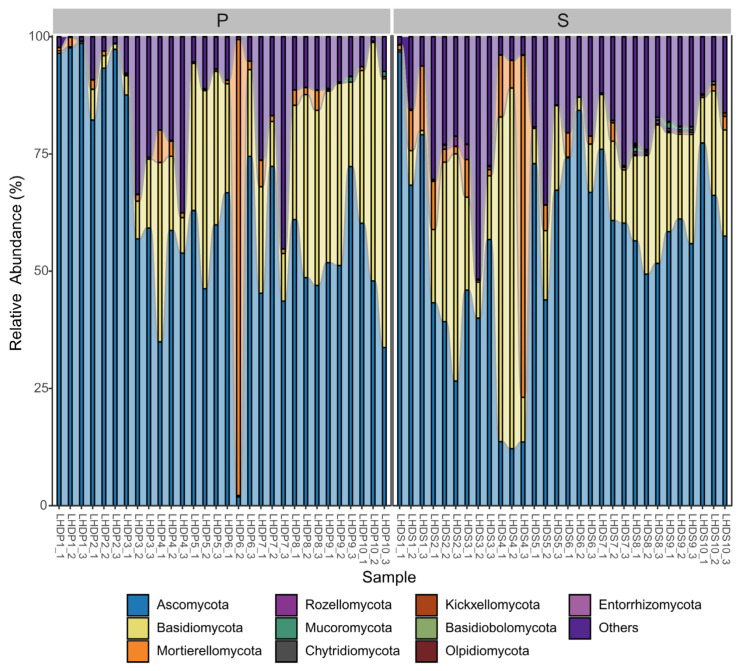
Relative abundance of phyla in the mycobiomes (top ten phyla) of weathered rocks (P) and sediments (S) in Luohandu cave, Guilin, southern China.

Totally, we detected 15 phyla in the mycobiomes of weathered rocks (n = 30) and sediments (n = 30), i.e., Ascomycota, Basidiomycota, Mortierellomycota, Rozellomycota, Mucoromycota, Chytridiomycota, Kickxellomycota, Basidiobolomycota, Olpidiomycota Entorrhizomycota, Glomeromycota, GS01, Neocallimastigomycota, Zoopagomycota and Aphelidiomycota. The relative abundances of the top 10 phyla are shown in Figure 2. The phylum Ascomycota dominated all samples (relative abundance: 1.87–98.55% in weathered rocks, 12.15–96.79% in sediments), followed by Basidiomycota (relative abundance: 0.31–57.26% in weathered rocks, 0.18–76.91% in sediments) and Mortierellomycota (relative abundance: 0.08–97.22% in weathered rocks, 0.05–72.94% in sediments) (Figure 2). Despite the differences within and between weathered rock and sediment samples, the three phyla mentioned above appeared to be the most abundant groups in Luohandu cave. The phylum Rozellomycota was only observed in five weathered rock samples, with relative abundances ranging from 0.02 to 0.43%. In contrast, it was detected in 67% of the sediment samples, with relative abundances ranging from 0.01 to 3.23%. The phyla Mucoromycota, Chytridiomycota, Kickxellomycota and Basidiobolomycota showed the lower relative abundances, which were under 1.13% and 1.29% for weathered rock and sediment samples, respectively (Figure 2). The phyla Olpidiomycota and Entorrhizomycota were only present in the sediment samples, with the lowest relative abundance, which was below 0.32% (Figure 2).

**Figure 3:**
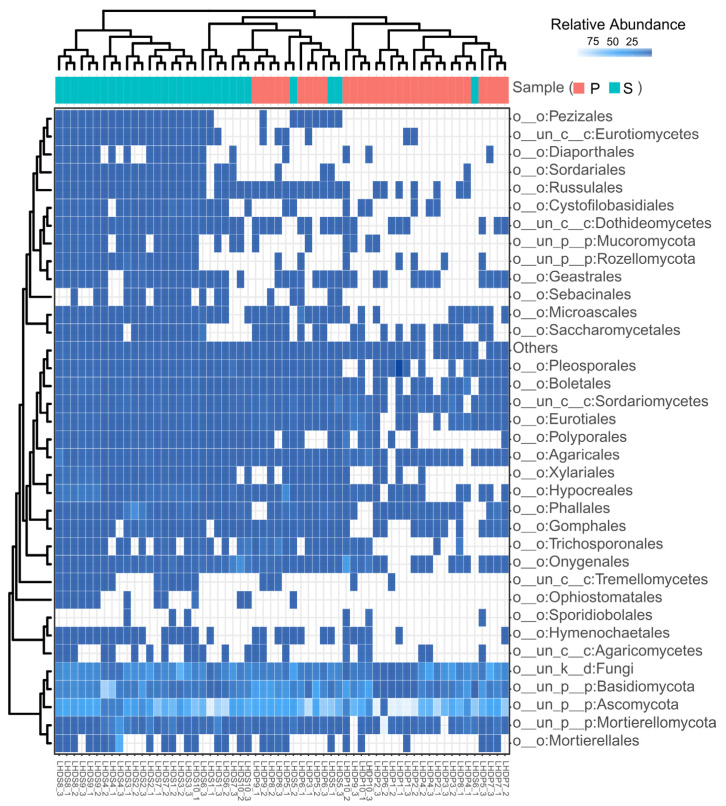
Heatmap of the 35 most abundant orders in the mycobiomes of Luohandu cave, Guilin, southern China.

At the order level, a total of 90 known orders and 26 unclassified groups were obtained for weathered rocks (n = 30) and sediments (n = 30) in Luohandu cave. The heat map in Figure 3 shows the clustering of the top 35 orders (24 classified and 11 unclassified orders) in the mycobiomes of 60 samples. Among the 24 classified orders, 11 belonged to five classes (Eurotiomycetes, Sordariomycetes, Pezizomycetes, Dothideomycetes and Saccharomycetes) of Ascomycota, 12 to three classes (Agaricomycetes, Tremellomycetes and Microbotryomycetes) of Basidiomycota, 1 to the class Mortierellomycetes of Glomeromycota. The mycobiomes basically clustered together according to the sample type and showed niche specificity (Figure 3). According to their average relative abundance, the top 15 classified orders were Onygenales (1.94%), Hypocreales (1.58%), Phallales (1.32%), Mortierellales (0.94%), Eurotiales (0.90%), Trichosporonales (0.84%), Agaricales (0.81%), Boletales (0.74%), Pleosporales (0.47%), Polyporales (0.45%), Xylariales (0.43%), Gomphales (0.39%), Microascales (0.32%), Geastrales (0.22%) and Cystofilobasidiales (0.19%), with the number of detected sequences ranging from 9020 to 80,813. They appeared mainly distributed in six classes of three phyla, i.e., Eurotiomycetes, Sordariomycetes and Dothideomycetes in Ascomycota, followed by Agaricomycetes and Tremellomycetes in Basidiomycota and Mortierellomycetes in Mortierellomycota.

Despite the orders of Onygenales, Hypocreales, Phallales, Mortierellales and Eurotiales being the top five classified orders in the mycobiomes of all samples, the predominant classified orders were not the same in the two types of cave samples (Figure 3). For weathered rocks, the top five classified orders based on their average relative abundances were Onygenales (1.88%), Eurotiales (1.11%), Trichosporonales (1.05%), Hypocreales (0.89%) and Agaricales (0.75%). In contrast, the most abundant classified orders for the sediment samples were Hypocreales (2.27%), Onygenales (1.99%), Phallales (1.95), Mortierellales (1.76%) and Agaricales (0.86%). Some groups within the phyla of Ascomycota, Basidiomycota and Mortierellomycota remained unclassified at the order level, which indicates the high mycobiome diversity of the Luohandu cave environments (Figure 3).

The mycobiomes in Luohandu cave appeared highly diverse, with 267 known genera (Table S1). However, at the genus level, 170 unclassified groups at the order, class, family and phylum levels were detected. The top five genera were *Mortierella*, *Myriodontium*, *Cutaneotrichosporon*, *Phallus* and *Rhizopogon*, with the number of detected sequences ranging from 27,585 to 39,195. Although we totally detected 120 and 240 known genera in weathered rocks and sediments (Table S1), only 93 known genera were shared by the two kinds of samples, and 27 and 147 unshared genera were found in weathered rocks and sediments, respectively.

**Figure 4:**
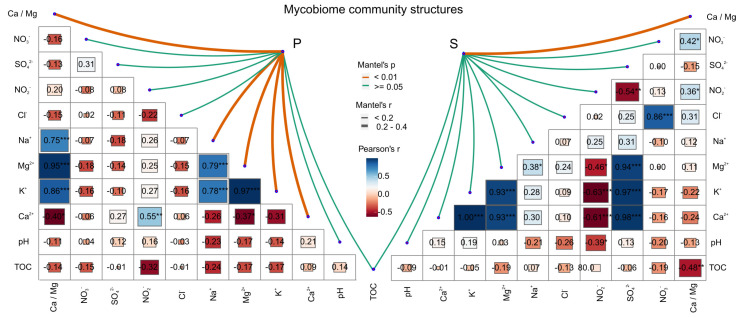
Correlations between mycobiome community structures and environmental variables in Luohandu cave, Guilin, southern China. *: *p* < 0.05; **: *p* < 0.01; ***: *p* < 0.001.

In terms of the structure of the mycobiome communities and environmental variables in general, we found that the environmental variables significantly affected the structure of the mycobiome community of weathered rocks (Mantel test, r = 0.3034, *p* = 0.001). In contrast, no significant relationship was found for the sediments (Mantel test, r = 0.0702, *p* = 0.182). We set out to further survey the main environmental variables that appeared to affect the structure of the mycobiome communities in weathered rocks or sediments. We found that the structure of the mycobiome community in weathered rocks exhibited strong correlations with the cations Ca^2+^ (Mantel test, r = 0.22, *p* = 0.001), K^+^ (r = 0.25, *p* = 0.001), Mg^2+^ (r = 0.27, *p* = 0.002) and Na^+^ (r = 0.22, *p* = 0.003) and with the Ca/Mg ratio (r = 0.24, *p* = 0.005) (Figure 4). For the sediments, however, a correlation was only revealed with the Ca/Mg ratio (Mantel test, r = 0.23, *p* = 0.003). Therefore, these results indicated that the Ca/Mg ratio is a major factor affecting the diversity and structure of mycobiome communities in weathered rocks and sediments in Luohandu cave (Figure 4).

### 3.3. Biodiversity Assessment of the Mycobiomes in Weathered Rocks and Sediments

**Figure 5:**
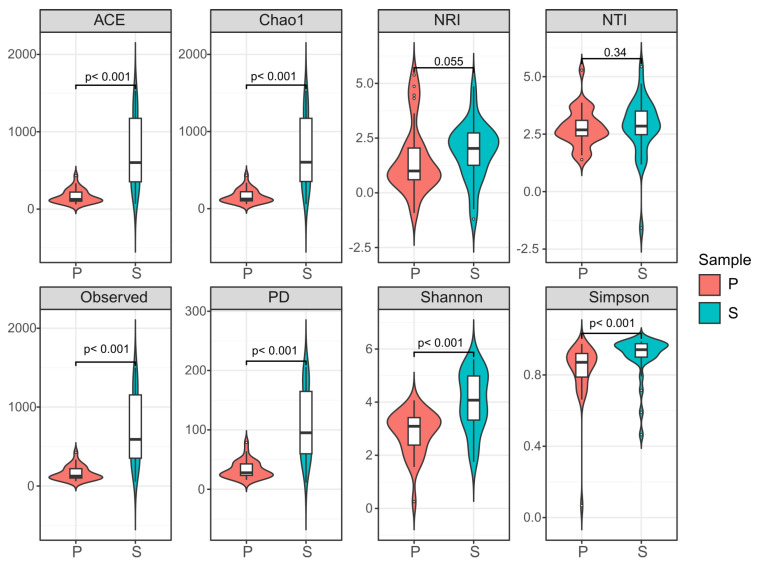
Comparison of the alpha diversity indices of the mycobiomes of weathered rocks (P) and sediments (S) in Luohandu cave, Guilin, southern China.

The alpha diversity indicated that the mycobiomes were highly diverse in weathered rocks and sediments in Luohandu cave. The number of species was fully captured by mycobiome sequencing, as evidenced by the high values of observed species (from 61.00 to 428.00 in weathered rocks and from 55.00 to 1498 in sediments) and Chao 1 metric (from 61.00 to 434.00 in weathered rocks and from 61.00 to 1528.71 in sediments, Wilcoxon’s test, *p* < 0.001, Figure 5). The richness metrics of observed-species, Chao 1 and ACE (Wilcoxon’s test, *p* < 0.001, Figure 5) showed that the community richness was significantly different between weathered rocks and sediments. The ACE index varied from 64.00 to 432.84 and from 67.00 to 1528.34 for weathered rocks and sediments, respectively. The Shannon index varied from 0.25 to 4.07, with an average of 2.85, for weathered rocks and from 1.76 to 5.61, with an average of 4.04, for the sediments, whilst the Simpson index ranged from 0.71 to 0.99, with an average of 0.87, for a total of 60 samples. The PD indices of weathered rocks and sediments were significantly different (Wilcoxon’s test, *p* < 0.001, Figure 5) and varied from 15.81 to 78.93, with an average of 33.00, and from 11.40 to 206.02, with an average of 107.98, for weathered rocks and sediments, respectively. Generally, the sediments possessed the relatively highest alpha diversity and were significantly different from weathered rocks, according to the diversity indices and richness metrics (Wilcoxon’s test, *p* < 0.001, Figure 5). The mycobiome lineage, however, showed an aggregation pattern, since the NTI index (0.34) and NRI index (0.055) were greater than zero (Figure 5).

**Figure 6:**
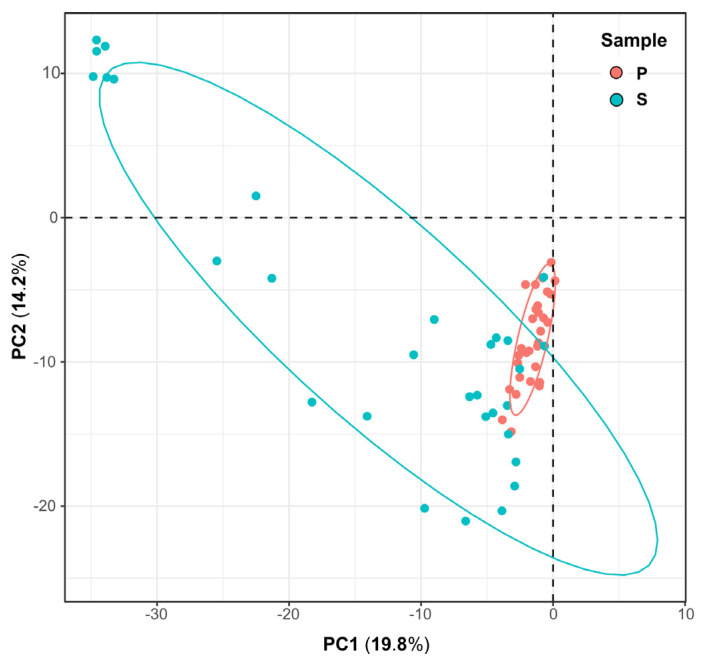
Principal component analysis (PCA) of the mycobiomes of weathered rocks (P) and sediments (S) in Luohandu cave, Guilin, southern China.

The results of beta diversity analysis showed a significant difference between weathered rocks and sediments. The mycobiome compositions of weathered rocks and sediments clearly clustered according to the habitats in principal component analysis (PCA, Figure 6). A PCA plot based on phylogenetic isometric log ratio transform showed the phylogenetic relationship between weathered rock and sediment samples. PC1 and PC2 explained 19.8% and 14.2% of the variation, respectively (Figure 6). The mycobiome communities in the sediments showed a dispersed distribution, while those in weathered rocks clustered well. However, a few exceptional samples did not cluster well, due to the heterogeneity of the mycobiomes in different samples (Figure 6).

**Figure 7:**
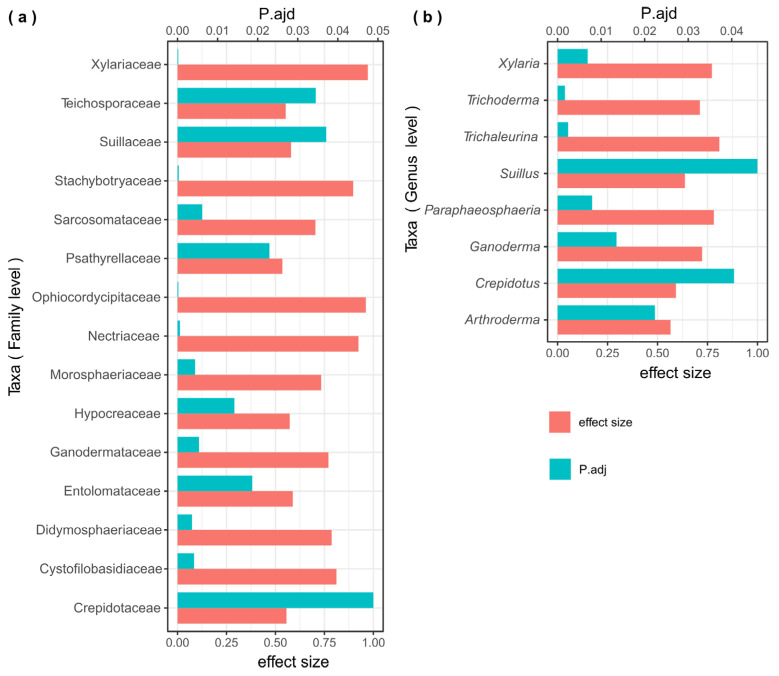
Results of the ALDEx2 analysis comparing the mycobiomes at the family (**a**) and genus (**b**) levels in Luohandu cave, Guilin, southern China. False discovery rate (FDR)-adjusted *p*-values below 0.05 (significant differences) and effect size values for each taxon are shown in (**a**,**b**).

The ALDEx2 analysis was combined with a differential abundance analysis for the comparison of two or more conditions. The analysis using ALDEx2 showed 15 identified families and eight identified genera with significant differences in the sediment samples (Wilcoxon’s test, false discovery rate (FDR)-adjusted *p*-values < 0.05, Figure 7a,b). No identified taxa at the family and genus levels with significant differences were found in the weathered rock samples. The mycobiome taxa (family level) with significant differences were mainly distributed in Ascomycota and Basidiomycota, that is, Hypocreaceae, Xylariaceae, Nectriaceae, Ophiocordycipitaceae, Didymosphaeriaceae, Stachybotryaceae, Sarcosomataceae, Morosphaeriaceae and Teichosporaceae in Ascomycota, Psathyrellaceae, Cystofilobasidiaceae, Suillaceae, Ganodermataceae, Crepidotaceae and Entolomataceae in Basidiomycota (Wilcoxon’s test, false discovery rate (FDR)-adjusted *p*-values < 0.05, Figure 7a). At the genus level, the taxa that showed significant differences were *Xylaria, Trichoderma*, *Trichaleurina*, *Paraphaeosphaeria*, *Arthroderma*, *Suillus*, *Ganoderma* and *Crepidotus* (Wilcoxon’s test, false discovery rate (FDR)-adjusted *p*-values < 0.05, Figure 7b).

### 3.4. Mycobiome Interactions in Weathered Rocks and Sediments

**Figure 8:**
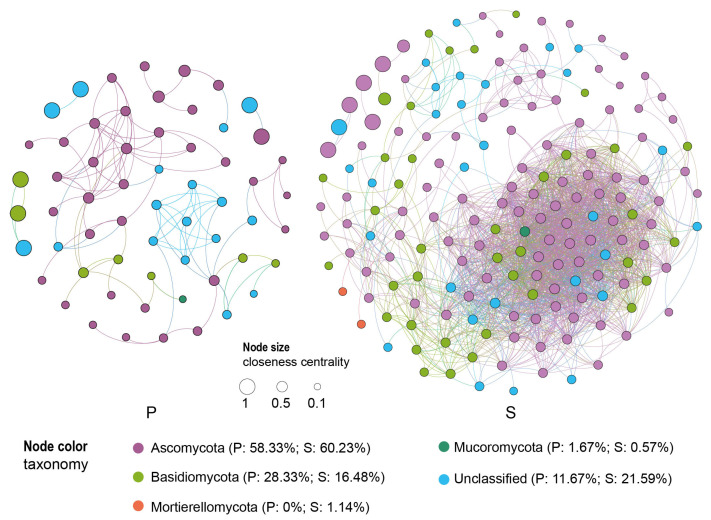
Co-occurrence networks of mycobiome communities based on pairwise Spearman’s correlations between ASVs, with cutoff of correlation coefficients of 0.641 (P) and 0.663 (S).

Co-occurrence network analysis unveiled the mycobiome interactions in weathered rocks and sediments. The network of weathered rocks demonstrated a significantly higher clustering coefficient of 0.54 (0.06, *p* < 0.01) and a shorter average path length of 1.97 (3.33, *p* < 0.01) compared to randomly generated networks from 100 simulations using the ER random network method. In contrast, the network of the sediments exhibited significantly higher values of the clustering coefficient (0.73 vs. 0.13, *p* < 0.01) and a similar average path length (2.31 vs. 1.88) compared to the random network (Figure 8). The results indicated that the mycobiome interactions in weathered rocks and sediments formed small-world networks. The mycobiome networks of weathered rocks and sediments exhibited modularity values of 0.67 and 0.23, respectively. Furthermore, the degree distribution of nodes in both networks followed a power law distribution (*p*: 0.30 vs. 0.99) according to the Kolmogorov–Smirnov test. The topological features of modularity, small-world network, and scale-free network indicated that the networks of weathered rocks and sediments were typical microbial networks. The network efficiency of weathered rocks was lower than that of the sediments based on the proportional removal of random nodes, suggesting that the sediment network exhibited greater robustness.

The co-occurrence network of weathered rocks consisted of 60 nodes and 102 edges, with a small percentage (11.76%) of them (12 edges) exhibiting a negative correlation. In contrast, the co-occurrence network of the sediments comprised 176 nodes and 2196 edges, with a lower proportion (1.10%) of edges (24 edges) exhibiting a negative correlation compared to the weathered rock network. The nodes in the co-occurrence network of weathered rocks were affiliated with three phyla, with Ascomycota representing the highest proportion (35 nodes, 58.33%), followed by Basidiomycota (17 nodes, 28.33%, Figure 8). In contrast, the nodes in the co-occurrence network of the sediments were associated with four phyla: Ascomycota occupied the highest number of nodes (106 nodes, 60.23%), followed by Basidiomycota (29 nodes, 16.48%), Mortierellomycota (2 nodes, 1.14%) and Mucoromycota (1 node, 0.57%). Naturally, the most significant interaction in the co-occurrence network of weathered rocks (54 edges, 52.94%) and sediments (1018 edges, 46.33%) was between Ascomycota and Ascomycota. The interaction between Ascomycota and Basidiomycota accounted for a high proportion (weathered rocks: 13 edges, 12.75%; sediments: 302 edges, 13.75%). In contrast, the interaction between Basidiomycota was significantly higher in the weathered rock network (18 edges, 17.65%) compared to the sediment network (29 edges, 1.32%). The result suggests a significant difference in the interaction patterns between weathered rocks and sediments.

Betweenness centrality quantifies the extent to which a node resides on the shortest paths between other pairs of nodes, thereby influencing the flow of information or resources in the network. On the other hand, closeness centrality measures the proximity of a node to other nodes and its ability to access information or resources. In the weathered rock network, 7 of the top 10 nodes with the highest values of betweenness centrality were attributed to Ascomycota, and 1 node was affiliated with Basidiomycota (Table S2). Furthermore, among the top 10 nodes with the highest values of closeness centrality, 4 nodes were associated with Ascomycota, and 4 nodes with Basidiomycota (Table S3). In the sediment co-occurrence network, Ascomycota accounted for 7 and 8 of the top 10 nodes with the highest values of betweenness centrality and closeness centrality, respectively. The findings suggest that Ascomycota exerts a controlling influence on the mycobiome co-occurrence network of the sediments, whereas both Ascomycota and Basidiomycota play fundamental roles in the mycobiome co-occurrence network of weathered rocks. In the network of weathered rocks, a total of six ASVs were identified as keystone species (Table S4). Among them, two ASVs were affiliated with Basidiomycota, and four ASVs were associated with Ascomycota. In the sediment network, a total of eight ASVs were identified as keystone species (Table S4). Among them, five ASVs belonged to Ascomycota, and three ASVs were categorized as unclassified fungi. These keystone ASVs were present at a low abundance level (<1.29%) in both weathered rock and sediment samples (Table S4).

**Figure 9:**
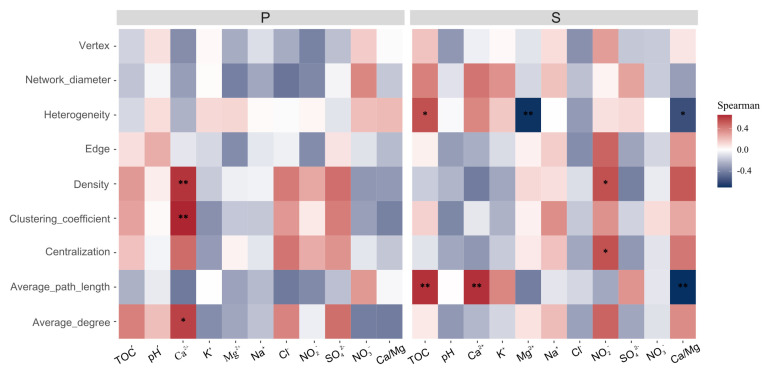
Linkages between co-occurrence network topological structures and environmental parameters. *: *p* < 0.05; **: *p* < 0.01.

The sub-networks obtained from the weathered rock network consisted of fewer nodes (25.3 ± 10.6 vs. 91.1 ± 39.1, *p* < 0.001) and edges (39.0 ± 16.9 vs. 890.0 ± 806.1, *p* < 0.001) compared to the sub-networks extracted from the sediment network. The network diameter (3.60 ± 1.13, 5.93 ± 1.86, *p* < 0.001) and average path length (1.53 ± 0.36, 1.85 ± 0.44, *p* < 0.001) were significantly lower for the sub-networks of weathered rocks compared to those of the sediments. The topological indices of the sub-networks of weathered rocks significantly increased with an increase in the values of environmental factors, as observed for density (rho = 0.62, *p* = 0.01), clustering coefficient (rho = 0.66, *p* = 0.008) and average degree (rho = 0.59, *p* = 0.02) with Ca^2+^ cation concentration (Figure 9). Additionally, the topological indices of the sub-networks of the sediments significantly and positively correlated with environmental factors, such as heterogeneity (rho = 0.54, *p* = 0.04) and average path length (rho = 0.63, *p* = 0.01) with TOC, as well as density (rho = 0.54, *p* = 0.04) and centralization (rho = 0.54, *p* = 0.04) with NO_2_^-^ concentration (Figure 9). Furthermore, heterogeneity exhibited a significant negative correlation with Mg^2+^ cation concentration (rho = −0.69, *p* = 0.002), while heterogeneity (rho = −0.58, *p* = 0.02) and average path length (rho = −0.70, *p* = 0.002) increased with a decrease in the Ca/Mg ratio (Figure 9). These results suggest that the complexity of environmental parameters affected the mycobiome co-occurrence networks.

## 4. Discussion

### 4.1. Disclosure of Luohandu Cave’s Potential “Hidden Diversity” in Mycobiomes

In subterranean karst caves, substrates such as air, sediments, weathered rocks, guano, dripping water, bat skin/fur, bat bones, dung, wood, earthworm casts and insects have been commonly sampled for taxonomic and mycobiome studies [4,6,7,14,16]. In our survey, we found that the sediments had higher values of Chao 1 metrics, ACE index, Shannon index, observed-species an PD metric than the weathered rock samples. Therefore, the sediments possessed the relatively highest alpha diversity according to the diversity indices and richness metrics (Figure 5). These results align with those of a statistical analysis of cave fungi [6] and the 454 pyrosequencing in Kartchner Caverns [17]. Yet, some deep-cave sediments may be a valuable genetic resource and harbor biomarkers of both modern and old microorganisms [31,32]. Sediments are the most commonly studied cave substrate and yield the greatest number of fungal isolates [6]. However, our previous reports is inconsistent with the results of Luohandu cave [4], as it showed a higher mycobiome diversity in weathered rocks than in sediments. The possible reasons for this discrepancy are probably the remarkable heterogeneity of the mycobiomes at a local scale, seasonal differences and microclimate features that benefit the occurrence of specific fungi [1,33,34,35].

Although traditional isolation methods can detect a high number of microorganism strains, less than 8% of the available information regards fungi, making it difficult to reveal the whole “hidden diversity” of mycobiomes in caves [36]. Luohandu cave, as other subterranean cave ecosystems, is ubiquitously colonized by fungi, as indicated by the recovery of ITS1 genes from 60 samples. We found 267 known genera and 90 known orders in 15 phyla in the mycobiomes of weathered rock (n = 30) and sediment (n = 30) samples. Ascomycota dominated all samples, followed by Basidiomycota (Figure 2). This result aligns with those of cave mycology studies based on culture and non-culture methods [4,7,16,37,38]. Among the fungal taxa reported from caves and mines worldwide before 2013, Ascomycota (69.1%) and Basidiomycota (20%) were the dominant groups [6]. This, is also supported by previous microorganism isolation works [7,16,37,39] and mycobiome data [4,38] regarding karst caves obtained recently.

We further revealed the existence of 200 known fungal families in Luohandu cave. The top 10 families are Onygenaceae, Arthrodermataceae, Aspergillaceae and Hypocreaceae in Ascomycota, Trichosporonaceae, Phallaceae, Rhizopogonaceae, Clathraceae and Gomphaceae in Basidiomycota, and Mortierellaceae in Mortierellomycota. It is interesting to note that although the Trichocomaceae family was reported as the dominant family of Ascomycota in caves and mines worldwide [6], it had a relatively low abundance and was only present in 19 samples in our survey. Notably, for Onygenaceae, a family of Ascomycota, we detected the highest number of sequences in 83% of the samples, indicating its predominance in Luohandu cave. The order Onygenales comprises a diverse group of Ascomycota that are commonly found in terrestrial habitats worldwide. This finding is contrast with a previous report [6], indicating that high-throughput sequencing methods can reveal the diversity of cave mycobiomes more effectively. To date, 13 families of Ascomycota have been most frequently detected in solution caves by culture-dependent methods. Of them, 11 families were also detected in this study, and they only accounted for 5.5% of the 200 known families found in Luohandu cave. Moreover, we found that only two families (Arthrodermataceae and Hypocreaceae) of the top 10 families mentioned above were previously reported in carbonate caves, indicating a potential mycobiome diversity of karst cave ecosystems.

Although the phylum Ascomycota was dominant, as previously reported, some subdominant groups that were different from those found in other caves. Cave mycology surveys are historically based on cultivation approaches, the inherent bias of such techniques causing an incomplete view of cave mycobiome diversity. For example, of the top 10 families in Basidiomycota mentioned above, none of them were confirmed to be frequently present in karst caves worldwide, including Fomitopsidaceae, Psathyrellaceae, Mycenaceae and Polyporaceae [6]. Moreover, as shown by the 200 known families and more than 70 unclassified groups revealed in this study, modern molecular techniques are superior to the traditional cultivation approaches in deconstructing the dimensions of mycobiome fingerprints in subterranean karst caves. Therefore, the potential “hidden diversity” in mycobiomes in Luohandu cave was revealed.

### 4.2. The Ca/Mg Ratio Significantly Affects the Mycobiome Community Structure

Calcium and magnesium ions, as in other eukaryotic organisms, are essential for the growth of fungi and are involved in diverse functions [40,41,42]. We explored the correlations between the Ca/Mg ratio and the structure of the mycobiome communities in the two kinds of samples from Luohandu cave (Figure 4). The results of this analysis were generally inconsistent with previous observations that temperature, weathering index and SO_4_^2−^ concentration [23], rather than the Ca/Mg ratio, were the strongest factors shaping bacterial community diversity and structure in Luohandu cave. In the study of cave microbes, a wide range of environmental variables, such as pH, temperature, CO_2_ concentration, distance from the natural entrance and content of cations and anions in nutrients are commonly measured and have revealed that environmental variables such as proximity to the natural entrance [17], substrate and spatial variables [20], climate [22] and ventilation [43] are major determinants of mycobiome communities in karst caves. However, among prokaryotes, archaea are more affected by pH and ORP (oxidation–reduction potential) [44], whilst phosphorus-bearing minerals are critical for epipetreous bacterial communities [38]. Taken together, these reported results suggest that bacterial and fungal communities respond differently to environmental variables in karst caves. Although eleven environmental variables were measured, only the Ca/Mg ratio exhibited strong correlations with the structure of the mycobiome communities in the two examined niches, while the concentrations of Ca^2+^, K^+^, Mg^2+^ and Na^+^ cations were only critical in weathered rocks. Thus, this result suggests that the Ca/Mg ratio may be an important factor affecting the structure of the mycobiome community in the Luohandu cave.

In the light of the known environmental variables in caves reported in previous studies and their key role in regulating the mycobiome community structure, as discussed above, it is likely that the absence of homogeneous variables is essential and serves as a major determinant of mycobiome communities in all karst caves. Given the heterogeneity of karst caves and the complexity of the environmental variables, as well as the significant heterogeneity of mycobiomes at a local scale, introducing a limited number of parameters in one analysis may underestimate the influence of other factors and conceal the variations in the structure of the communities caused by operational factors. Therefore, the structure of mycobiome communities in karst caves should be analyzed considering more environmental variables including those inside and outside the caves.

### 4.3. Ascomycota Dominate the Mycobiome Networks

We found that the degree distribution of the mycobiomes’ co-occurrence networks of weathered rocks and sediments in Luohandu cave followed a power law, as usually observed for peat sediment and soil microbial interaction networks [29,45]. The power law distribution was evidenced by analyzing competition, habitat filtering, evolution history and neutral process [46]. In our survey, few negative edges in the mycobiome-associated network suggested that habitat filtering and evolution history, rather than competition, had a strong influence on mycobiome interactions in weathered rocks and sediments of Luohandu cave. The increased number of nodes and edges in the co-occurrence network of the sediments suggests that the interactions within the mycobiome are tighter compared to those within the mycobiome of weathered rocks. This difference may be attributed to variations in energy dynamics between the two habitats.

In the co-occurrence network of weathered rocks, Ascomycota and Basidiomycota were the dominant taxa, with 35 and 17 nodes and 75 and 36 edges, respectively. However, in the network of the sediments, Ascomycota exclusively dominated, with 106 nodes and 1941 edges. Additionally, in the co-occurrence network of weathered rocks, four keystone ASVs were affiliated with Ascomycota, while two keystone ASVs were affiliated with Basidiomycota. In contrast, all five identified keystone ASVs in the sediment co-occurrence network were affiliated with Ascomycota. These keystone species maintained a relatively low abundance in both weathered rock and sediment samples, indicating the vulnerability of the Luohandu cave ecosystem and the disproportionate negative impact that would be caused by their removal [47]. According to the growth process of scale-free networks, keystone species are typically considered initial components of a network [48]. Therefore, Ascomycota and Basidiomycota may have experienced a long evolutionary history in weathered rocks, while Ascomycota may have undergone a prolonged period of evolution in the sediment habitat of Luohandu cave.

### 4.4. Limitations and Future Investigations

As a result of the advances in the high-throughput sequencing technology, we have now scratched the surface of the mycobiome structure in subterranean karst cave systems; however, the high level of mycobiome diversity that has been revealed so far has shown that much more work remains to be done in deconstructing the dimensions of the mycobiome fingerprints in the context of global changes. Generally, the distribution of mycobiomes in subterranean caves is influenced by host material (wood, guano, bat fur/skin, rock and sediments), micro-environmental conditions (water availability, temperature, pH and nutrient sources), cave-specific parameters (mineral composition, porosity and rock permeability) and constraints caused by the remote location of certain caves, the inaccessibility of specimens and technical limitations [4,6,22]. Our results may have been affected by multiple factors aforementioned, and more detailed sampling of various materials as well as seasonal variations should be considered in the near future. However, it should be noted that the structure of mycobiome communities should be analyzed introducing more environmental variables, due to the fact that considering a limited number of environmental factors can lead to one-sided conclusions. Therefore, it is worthwhile to consider multiple internal and external factors, such as overlying strata, niche variation and environmental physicochemical factors, and other influencing factors, such as microclimatic conditions, spatial variables and human influence, as a whole, and determine the dominant factors affecting the mycobiome community distribution and ecology of karst cave systems in the near future.

## 5. Conclusions

We systematically studied the mycobiome of weathered rocks and sediments in Luohandu cave (Guilin, Southern China) via high-throughput sequencing methods. We revealed 267 known genera and 90 known orders in 15 phyla in the mycobiomes of weathered rocks (n = 30) and sediments (n = 30). We revealed Ascomycota as the dominant phylum in all samples, followed by Basidiomycota and Mortierellomycota. We acknowledged that sediments possessed the relatively highest alpha diversity and were significantly different from weathered rocks according to the diversity indices and richness metrics. Fifteen families and eight genera with significant differences were detected in the sediment samples. The Ca/Mg ratio appeared to significantly affect the mycobiome communities’ structures. Ascomycota appeared to exert a controlling influence on the mycobiome co-occurrence network of the sediments, whilst Ascomycota and Basidiomycota were found to play fundamental roles in the mycobiome co-occurrence network of weathered rocks. Taken together, our results provide a more comprehensive description of the mycobiome fingerprints in Luohandu cave and offer a new window into the mycobiome communities and the ecology of subterranean karst cave ecosystems.

## Data Availability

The mycobiome sequencing data presented in this study are openly available in the National Omics Data Encyclopedia (NODE) at http://www.biosino.org/node, accessed on 21 December 2023, under the project accession OEP004875 and OEP004876.

## Supplementary Materials

The following supporting information can be downloaded at https://www.mdpi.com/article/10.3390/microorganisms12010211/s1, Table S1: The identified genera of the mycobiomes in Luohandu cave, southern China; Table S2: Top 10 nodes with the highest betweenness centrality in the mycobiome networks of Luohandu cave, southern China; Table S3: Top 10 nodes with the highest closeness centrality in the mycobiome networks of Luohandu cave, southern China; Table S4: Keystone species identified in the mycobiome networks of Luohandu cave, southern China.
