# Supplementary material for: Deconstructing the Dimensions of Mycobiome Fingerprints in Luohandu Cave, Guilin, Southern China
Source: Microorganisms. 2024 Jan 20;12(1):211. doi: 10.3390/microorganisms12010211 (PMC10818497; doi:10.3390/microorganisms12010211)
Supplement: Supplementary file 1 [file microorganisms-12-00211-s001.zip › Supplementary Materials.pdf]

Deconstructing the Dimensions of Mycobiome Fingerprints in Luohandu Cave, Guilin, Southern China

Bai-Ying Man [1,2,*], Xing Xiang [1,2], Xiao-Yu Cheng [3,4], Hong-Mei Wang[3,4,*], Chun-Tian Su [5,6], Qi-Bo Huang [5,6], Yang Luo [1,2], Chao Zhang [1,2], Gang Cheng [1,2], Yu-Yang Ni [1,2] and Xing-Hua Shao [1,2]

1 College of Life Science, Shangrao Normal University, Shangrao, China
2 Key Laboratory for Regional Plants Conservation and Ecological Restoration of Northeast Jiangxi, Shangrao Normal University, Shangrao, China; shmilying@126.com (B.-Y.M.), xiangxing1989@126.com (X.X.), 312144@sru.edu.cn (Y.-Y.N.), zhangchao_20180604@163.com (C.Z.), enjoy123fly@163.com (G.C.), yangl1829848@163.com (Y.L.), xinghuashao@126.com (X.-H.S.)
3 School of Environmental Studies, China University of Geosciences, Wuhan, China; chengxy@cug.edu.cn (X.-Y.C.), wanghmei04@163.com (H.-M.W.).
4 State Key Laboratory of Geobiology and Environmental Geology, China University of Geosciences, Wuhan, China
5 Institute of Karst Geology, CAGS/Key Laboratory of Karst Dynamics, MNR & GZAR, Guilin, China; schuntian@mail.cgs.gov.cn (C.-T.S.), qbohuang0108@163.com (Q.-B.H.).
6 Pingguo Guangxi, Karst Ecosystem, National Observation and Research Station, Pingguo, Guangxi, China
* Correspondence: shmilying@126.com (B.-Y.M.); wanghmei04@163.com (H.-M.W.)

Supplementary Materials:

Tabel S2 Top 10 nodes with the highest betweenness centrality in mycobiome networks of Luohandu cave, southern China

Weathered rock networks			Sediments networks		
**Lable**	**Phylum**	**Betweenness centrality**	**Label**	**Phylum**	**Betweenness centrality**
ASV1663	Ascomycota	124.6	ASV7554	Ascomycota	1833.1
ASV1833	Unclassified	102.0	ASV8525	Ascomycota	1547.1
ASV8564	Ascomycota	85.2	ASV7543	Unclassified	1503.9
ASV3404	Basidiomycota	64.0	ASV2556	Basidiomycota	1329.4
ASV8545	Ascomycota	62.6	ASV5140	Ascomycota	1279.1
ASV8485	Unclassified	56.0	ASV3586	Basidiomycota	1157.6
ASV6757	Ascomycota	51.0	ASV6522	Ascomycota	1133.4
ASV8134	Ascomycota	46.0	ASV2139	Ascomycota	1105.9
ASV2246	Ascomycota	43.7	ASV8545	Ascomycota	993.6
ASV6760	Ascomycota	39.0	ASV7572	Ascomycota	928.4

Tabel S3 Top 10 nodes with the highest closeness centrality in mycobiome networks of Luohandu cave, southern China

Weathered rock networks			Sediments networks		
Lable	Phylum	Closeness centrality	Label	Phylum	Closeness centrality
ASV3504	Basidiomycota	1.00	ASV191	Ascomycota	1.00
ASV3510	Basidiomycota	1.00	ASV2405	Ascomycota	1.00
ASV4048	Basidiomycota	1.00	ASV4220	Basidiomycota	1.00
ASV4300	Basidiomycota	1.00	ASV6403	Ascomycota	1.00
ASV6290	Unclassified	1.00	ASV6745	Ascomycota	1.00
ASV6452	Ascomycota	1.00	ASV6814	Ascomycota	1.00
ASV8494	Unclassified	1.00	ASV6687	Unclassified	0.67
ASV1253	Ascomycota	0.63	ASV6760	Ascomycota	0.67
ASV1258	Ascomycota	0.63	ASV6922	Ascomycota	0.45
ASV8545	Ascomycota	0.54	ASV898	Ascomycota	0.45

Table S4 Keystone species identified from mycobiome networks of Luohandu cave, southern China

Samples	Label	Phylum	Relative abundance (%)	Degree	Closeness centrality	Betweeness centrality
Weathered rocks	ASV4327	Basidiomycota	0.53	5	0.392	7.17
	ASV4703	Basidiomycota	0.14	6	0.426	13.00
	ASV8527	Ascomycota	0.17	5	0.403	0
	ASV8540	Ascomycota	0.35	8	0.455	15.10
	ASV8544	Ascomycota	1.29	8	0.455	4.80
	ASV8563	Ascomycota	0.49	6	0.424	0
Sediments	ASV375	Ascomycota	0.06	40	0.374	10.90
	ASV2107	Ascomycota	0.05	29	0.364	12.00
	ASV4438	Unclassified	0.09	35	0.377	10.70
	ASV4455	Unclassified	0.25	40	0.373	5.41
	ASV4917	Ascomycota	0.06	33	0.370	1.27
	ASV6188	Unclassified	0.08	37	0.353	2.35
	ASV7751	Ascomycota	0.08	26	0.360	5.08
	ASV7979	Ascomycota	0.07	17	0.359	5.88
